# WGCNA combined with machine learning to explore potential biomarkers and treatment strategies for acute liver failure, with experimental validation

**DOI:** 10.1016/j.iliver.2024.100133

**Published:** 2024-11-13

**Authors:** Xinyan Wu, Xiaomei Zheng, Gang Ye

**Affiliations:** College of Veterinary Medicine, Sichuan Agricultural University, No. 211 Huimin Road, Wenjiang District, Chengdu 611130, China

**Keywords:** Acute liver failure, Machine learning, Eriodictyol, Potential biomarker, Weighted gene co-expression network analysis, Differential gene expression analysis

## Abstract

**Background and aims:**

To identify biomarkers to predict acute liver failure and investigate the mechanisms and immune-related pathways linked to its onset and progression.

***Methods*:**

We analyzed gene expression differences between patients with acute liver failure (ALF) and controls in the GSE14668 dataset. Clinically relevant modules and key ALF-associated genes were identified using weighted gene co-expression network analysis (WGCNA) in conjunction with differential gene expression (DEG) analysis. Enrichment analysis was carried out and protein–protein interaction networks were constructed to understand the functions and pathways. Six potential diagnostic biomarkers were identified using machine learning algorithms. Diagnostic performance was assessed via column charts and area under the curve calculations. Single-sample gene set enrichment analysis evaluated the relationship between known marker gene sets and potential biomarker expression. We also examined diagnostic biomarker mRNA levels in ALF models *in vivo* and *in vitro*. We estimated the relative infiltration levels of 22 immune cell subpopulations in ALF samples, and explored the link between diagnostic biomarkers and infiltrating immune cells.

***Result*:**

We found 352 DEGs associated with ALF. WGCNA analysis and intersecting DEGs identified 191 significant ALF-related genes. Machine learning identified *HORMAD2*, *WNT10A*, *ATP6V1E2*, *CMBL*, *ARRDC4*, and *LPIN2* as potential diagnostic biomarkers. Cell experiments and quantitative real-time polymerase chain reaction supported the therapeutic potential of eriodictyol for ALF. Immune infiltration analysis suggested that plasma cells, CD4 memory resting and activated T cells, macrophages, and neutrophils might play roles in the progression of ALF.

***Conclusion*:**

We identified *HORMAD2*, *WNT10A*, *ATP6V1E2*, *CMBL*, *ARRDC4*, and *LPIN2*, as diagnostic biomarkers for ALF and demonstrated the effectiveness of eriodictyol for treating ALF. Immune cell infiltration may play a significant role in the pathogenesis and progression of ALF.

## 1 Introduction

Acute liver failure (ALF) is a severe clinical syndrome with a high mortality rate of up to 80%, characterized by rapid progression, numerous complications, and high fatality [1,2]. ALF arises from liver injury induced by various stimuli, including viral agents, pharmaceuticals, alcohol consumption, and genetic metabolic aberrations [3]. It is frequently concomitant with systemic multiple organ failure and inflammatory response, which may contribute to its swift progression and resulting death [4]. The etiopathogenesis of ALF is marked by an immune-mediated inflammatory response [5]. Given the high mortality rate and complications linked to ALF, there is an urgent need to explore effective pharmacological interventions and gain a thorough understanding of its pathogenesis. Despite extensive efforts however, there is still a lack of effective treatment strategies for ALF, with liver transplantation remaining the main treatment option [6]. There is thus an urgent need to understand the pathogenesis of the disease and conduct targeted therapy, to provide the basis for subsequent in-depth basic research aimed at improving the prognosis of patients with ALF.

Recent progress in bioinformatics has witnessed the advent of novel and comprehensive analytical methodologies, predominantly relying on microarray technologies. These advancements have allowed the detection of pivotal genes related to specific disease states, thereby furthering the potential for diagnosis through biomarker identification [7,8]. Machine learning algorithms have found extensive medical applications, including Least Absolute Shrinkage and Selection Operator (LASSO) models and Support Vector Machine (SVM)-Recursive Feature Elimination (RFE) analysis [9,10]; however, limited studies have explored the application of machine learning techniques to identify biomarkers in ALF. Weighted gene co-expression network analysis (WGCNA) is a powerful algorithm available in the R package that allows the extraction of meaningful module information from gene expression data. By analyzing congruent expression patterns among various genes within a sample, WGCNA can categorize them into co-expression networks or modules, based on the correlation coefficients between genes [11]. WGCNA has been used in medical research to detect target genes in the therapeutic context of glioblastoma [12], Alzheimer’s disease, and osteoporosis [13]. WGCNA is thus a valuable technique for mining genes of interest. Eriodictyol is a potent candidate drug with extensive pharmacological activity and robust antioxidant properties, which has shown promising efficacy for treating ALF and offering liver protection. We previously showed that eriodictyol alleviated lipopolysaccharide (LPS)/d-galactosamine (D-GalN)-induced acute liver injury by inhibiting oxidative stress and cell apoptosis via regulating the PI3K/Akt signaling pathway. Eriodictyol has also demonstrated the ability to mitigate hepatotoxicity induced by various factors, such as acetaminophen and arsenic trioxide [14,15]. These results warrant further investigations into the potential of eriodictyol as a viable treatment for ALF, to enhance patient prognosis.

In this study, we carried out differential gene expression analysis (DEG) using a dataset of patients with ALF obtained from the Gene Expression Omnibus (GEO) database. We additionally employed WGCNA to identify gene modules that were co-expressed with the ALF phenotype and developed a machine learning model to locate potential biomarkers of ALF. The diagnostic efficacy of these potential biomarkers was evaluated using receiver operating characteristic (ROC) curve analysis. We also validated the effectiveness of eriodictyol for treating ALF using *in vitro* cell experiments and quantitative real-time polymerase chain reaction (qRT-PCR) to confirm the expression of potential biomarkers. Furthermore, we assessed the relationship between immune cells and ALF to provide valuable insights for future research.

## 2 Materials and methods

### 2.1 Bioinformatics analysis

Please refer to the supplementary documents for specific methods.

### 2.2 Animal study

#### 2.2.1 Animals and experimental design

Animal research was carried out according to the ethical guidelines approved by the Institutional Animal Care and Use Committee of the Institutions and Research Center of Sichuan Agricultural University. The principles of randomization and non-blinding were adhered to throughout. Male ICR mice, 64 weeks old, weighing (20 ± 2) g, were acquired from Chengdu Dashuo Biotechnology Co., Ltd. The mice were housed in a controlled environment with constant temperature (23 ± 2) °C and relative humidity (55% ± 5%), following a 12-h light–dark cycle, with *ad libitum* access to food and water. After 7 days of adaptive feeding, the mice were divided randomly into six groups (*n* = 10 per group): control group (normal saline), model group (LPS/D-GalN), positive drug group (LPS/D-GalN + silibinin 100 mg/kg), low-dose group (LPS/D-GalN + eriodictyol 10 mg/kg), medium-dose group (LPS/D-GalN + eriodictyol 20 mg/kg), and high-dose group (LPS/D-GalN + eriodictyol 40 mg/kg). Silibinin, eriodictyol, and normal saline were administered by intraperitoneal injection once a day for 7 days. One hour after administration on the 7th day, the mice in the LPS/D-GalN, LPS/D-GalN + eriodictyol (10, 20, and 40 mg/kg), and LPS/D-GallN + silibinin groups were injected intraperitoneally with 600 mg/kg D-GalN and 10 μg/kg LPS, while mice in the control group received a saline injection. After 6 h, the mice were euthanized by cervical dislocation and their livers were removed and stored at −80 °C for subsequent experiments.

#### 2.2.2 qRT-PCR

Total RNA was extracted from the liver using TRIzol reagent (Transgene, Beijing, China) and then converted into cDNA using a reverse transcription kit (Transgene). Fluorescence qRT-PCR was carried out using SYBR Green (Transgene, Beijing, China). The primer sequences are listed in Table 1. QX400 (Sichuan Jielaimei Technology Co., Ltd) was used for qPCR. Relative expression levels were measured and standardized using the 2^−ΔΔCq^ method, with glyceraldehyde 3-phosphate dehydrogenase (GAPDH) as the internal reference gene.

**Table 1:** Primer list.

Gene	Primer sequence Forward (5′–3′)	Reverse (5′–3′)
Lpin2	TGCCTTTCCATGCCCAGAGTTC	AGGTCATCCAGGTCCAGGTCAG
Arrdc4	AAGTTCCAGATCAGAGCGTCAGAC	ATAGGAGTCAATAAGGGCGGTGTG
Cmbl	ATCGGGAGGTTGATGCTGTCTTG	GCCCACAATGCCAATCTTCTGAG
GAPDH	CATCCGTAAAGACCTCTATGCCAAC	ATGGAGCCACCGATCCACA

### 2.3 Cell culture

HepG2 human liver cancer cells were obtained from Shanghai Fuheng Cell Center and cultured in an incubator at 37 °C, 5% CO_2_, and 95% humidity with 10% fetal bovine serum, 1% penicillin and streptomycin, and 1% HEPES buffer.

#### 2.3.1 Cell viability assay

HepG2 cells were seeded at a density of 10^4^/mL in a 96-well plate and incubated at 37 °C with 5% CO_2_ for 24 h. The culture medium was subsequently changed to medium containing eriodictyol at varying concentrations (12.5, 25, 50, 100, 200 μM) followed by further incubation for 24 h. Cell viability was assessed utilizing a CCK-8 assay kit (Solarbio, China). HepG2 cells in logarithmic growth phase were then cultured in a 96-well plate at 37 °C and 5% CO_2_ for 24 h. After discarding the supernatant, varying concentrations of D-GalN (2.5, 5, 10, 15, 20 mg/mL) combined with LPS (1 μg/mL) were introduced. The cells were then incubated for an additional 24 h, and their viability was subsequently quantified. Finally, HepG2 cells were seeded at a density of 10^4^/mL in a 96-well plate and incubated at 37 °C with 5% CO_2_ and then exposed to eriodictyol at different concentrations (12.5, 25, 50 μM) for 24 h, followed by concurrent treatment with D-GalN and LPS for an additional 24 h. Post-treatment, each well received 10 μL of CCK-8 solution and was further incubated for 1.5 h at 37 °C, and the absorbance of each well at 450 nm was measured using a microplate reader (Thermo Fisher Scientific Co., Ltd.).

#### 2.3.2 Alanine aminotransferase (ALT) and aspartate aminotransferase (AST)

Cells were co-treated with eriodictyol and LPS/D-GalN as described above. ALT and AST levels in the cell culture supernatant were then assessed utilizing ALT and AST assay kits (Nanjing Jiancheng Bioengineering Institute, Nanjing, China), according to the manufacturer’s instructions.

#### 2.3.3 Intracellular reactive oxygen species (ROS)

HepG2 cells were co-treated with eriodictyol and LPS/D-GalN as described above and intracellular ROS levels were quantified using a DCFH-DA kit (Solarbio, China). Following LPS/D-GalN treatment for 24 h, the cells were stained according to the kit instructions and incubated for a further 20 min. The resulting fluorescence was observed using a fluorescence microscope.

#### 2.3.4 Mitochondrial membrane potential (MMP)

Cells were co-treated with eriodictyol and LPS/D-GalN as described above and then processed using a JC-10 assay kit (Solarbio, China), according to manufacturer’s instructions. Finally, the cells were resuspended in PBS and observed under a fluorescence microscope.

#### 2.3.5 qRT-PCR

HepG2 cells were seeded at a density of 10^4^/mL in a 6-well plate and cultured at 37 °C and 5% CO_2_ for 24 h, followed by pretreatment with eriodictyol (0, 12.5, 25, 50 μM/L) for 24 h. The cells were then exposed to LPS/D-GalN treatment for another 24 h and subsequently harvested. Total RNA was isolated using TRIzol reagent (Transgene) and converted into cDNA using a reverse transcription kit (Transgene). qRT-PCR was performed using SYBR Green (Transgene). The primer sequences are provided in Table 2. Relative expression levels were determined using the 2^−ΔΔCq^ method, with GAPDH as the internal reference gene.

**Table 2:** Primer list.

Gene	Primer sequence Forward (5′–3′)	Reverse (5′–3′)
LPIN2	TGCCACCTCACCAATTCCTACTG	TCTCCACCCGATTTCACCAAAGG
ATP6V1E2	CGATGCCAAGGCTGAGGAAGAG	TTCTGCTGCTCTATCTGCTTCTCC
CMBL	GGGATTCTGCTGGGGTGGAAC	CTTGACAATGCCATAGACGGACAC
GAPDH	ACAACTTTGGTATCGTGGAAGG	GCCATCACGCCACAGTTTC

### 2.4 Immune infiltration analysis

We analyzed the composition of 22 invasive immune cells in the GSE14668 dataset samples using the CIBERSORT algorithm (https://cibersort.stanford.edu/). We then conducted additional comparative analyses to assess the distribution of immune cells between the ALF and normal groups.

### 2.5 Statistical analysis

Statistical tests, including *t*-tests and analysis of variance, were conducted using GraphPad Prism 9.0 (GraphPad Software, La Jolla, CA, USA). The results were presented as mean ± standard deviation. A *p*-value < 0.05 was considered statistically significant.

## 3 Results

### 3.1 DEG and GSEA

**Fig. 1:**
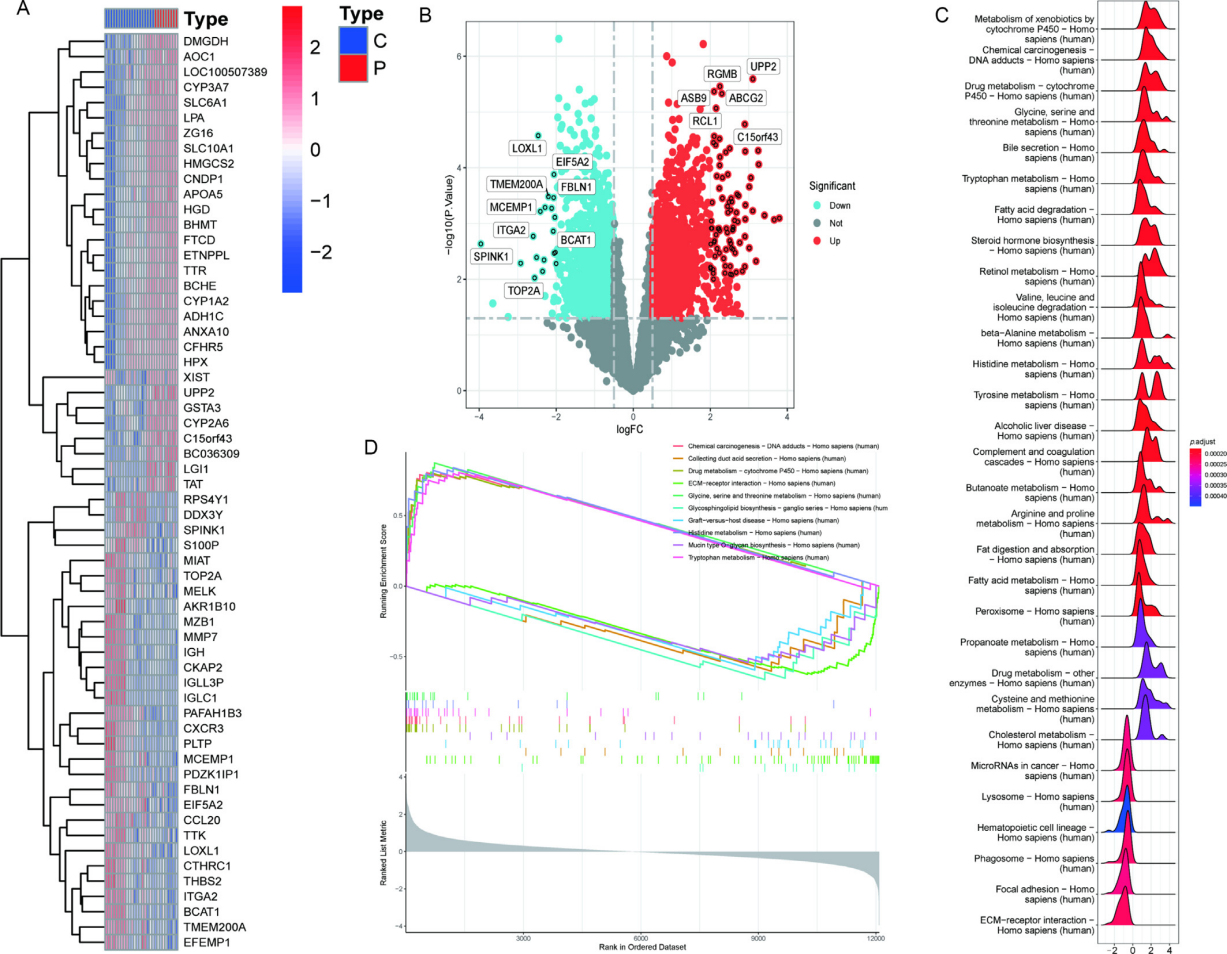
DEG and GSEA. (A) Differential gene heat map of GSE14668. Red: high expression; blue: low expression. (B) Differential gene volcano map of GSE14668. Red: upregulated; blue: downregulated; gray: no significant difference. Fold change is indicated. (C) GSEA differential pathway analysis for GSE14668. (D) Top five upregulated pathways and top five downregulated pathways.

Using the GSE14668 dataset, this study identified 352 DEGs, including 237 upregulated and 115 downregulated genes (Fig. 1A, B). We investigated the disparities in biological functions between ALF patients and normal samples by GSEA and revealed 30 significant pathways, including 24 upregulated and six downregulated pathways (Fig. 1C). The top five pathways are displayed. Upregulated pathways included Drug metabolism cytochrome P450, Glycine, serine, and threonine metabolism, and Histidine metabolism (Fig. 1D), and downregulated pathways included Collecting product acid secretion, ECM-receptor interaction, and Glycosphingolipid biosynthesis.

### 3.2 WGCNA and gene screening

**Fig. 2:**
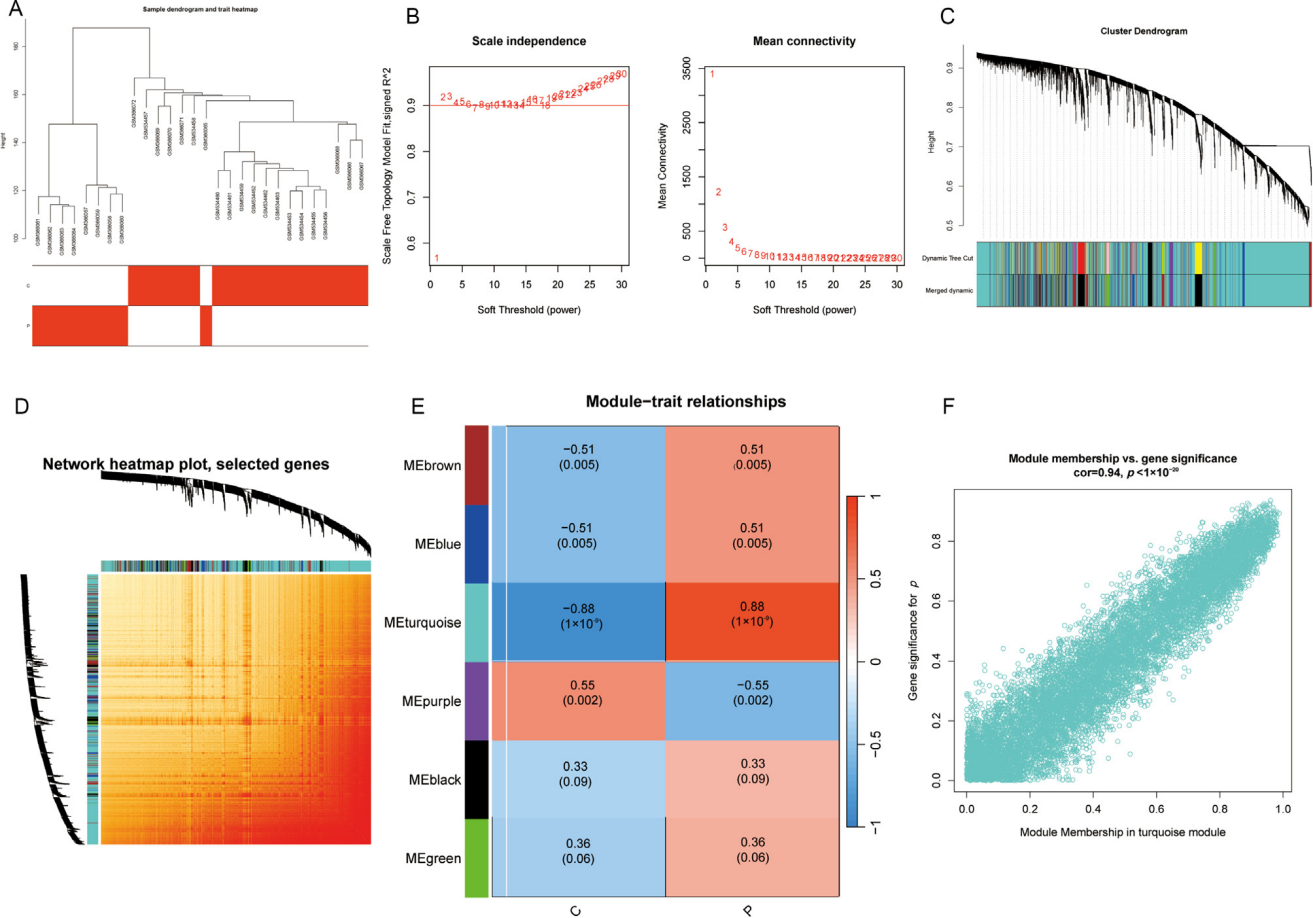
WGCNA. (A) Sample tree, with each branch representing a sample. Height on the vertical axis indicates clustering distance; horizontal axis represents clinical grouping information. (B) Soft threshold power using the scale-free fitting index (left) and average connectivity (right). (C) Tree clustering diagram generated by performing average chain hierarchical clustering of identified modules. Horizontal axis represents different genes and vertical axis shows correlation between genes. Lower branches indicate a stronger correlation between the genes within the branch. (D) Heat map illustrating topological overlap matrix (TOM) of genes selected for weighted co-expression network analysis. Light colors: lower overlap; red: higher overlap. (E) Heat map depicting correlation between module characteristic genes and clinical traits of ALF. (F) Scatter plot showing significance of turquoise module and ALF gene.

**Fig. 3:**
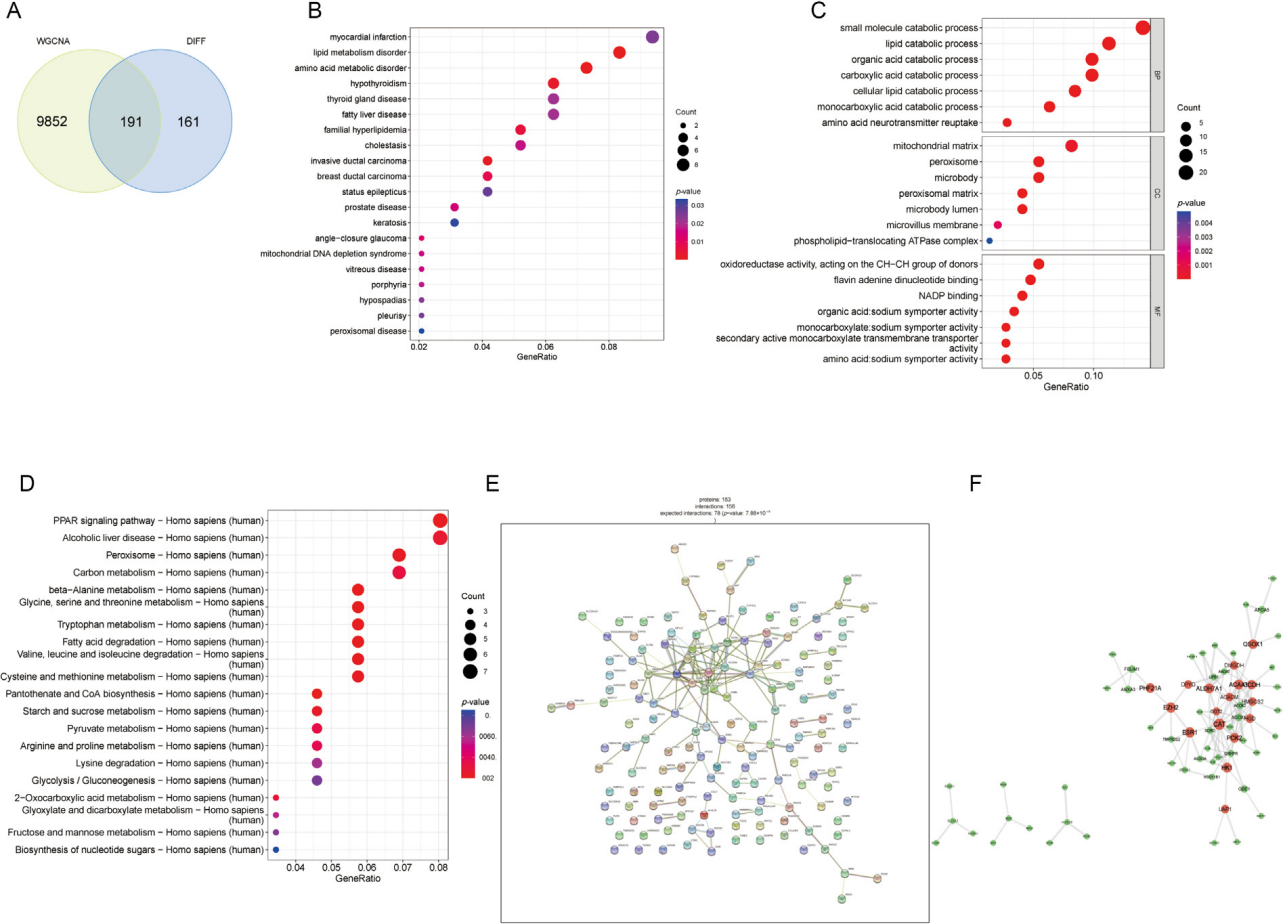
PPI network and enrichment analyses. (A) Intersection of DEG and WGCNA emerald module genes in the GSE14668 dataset. (B) GO enrichment analysis of 191 intersecting genes (top 7 BP, CC, MF). (C) Most important KEGG pathways in the top 20. (D) Top 20 most significant DO analyses. (E–F) PPI network analysis for the 191 intersecting genes.

Clustering analysis of all GSE14668 samples showed distinct clustering between samples (Fig. 2A). Hierarchical clustering analysis of the samples showed good clustering between the samples with no exclusions from the cluster tree view. To determine the adjacency relationship, we calculated the soft threshold power and increased the co-expression similarity using the pickSoftThreshold function in WGCNA, performing network topology analysis. Setting the soft threshold power to 6 (Fig. 2B), we identified six modules (Fig. 2C, D) through average hierarchical clustering and dynamic tree pruning. The turquoise module (Fig. 2E) exhibited a substantial correlation with ALF (*R* = 0.88, *p* < 0.001), making it a pivotal candidate for further analysis. Figure 2F shows a scatter diagram of ALF module membership and gene significance. We subsequently identified 191 ALF disease genes through intersection of the 10,043 genes in the turquoise module with the 352 DEGs, paving the way for subsequent analysis (Fig. 3A).

### 3.3 GO, DO, and KEGG enrichment analysis and PPI network construction

We investigated the biological functions of 191 ALF disease genes by GO, DO, and KEGG enrichment analyses. GO enrichment analysis revealed the biological processes (BP), molecular functions (MF), and cellular components (CC) of these genes, and the top seven were visualized using bubble charts (Fig. 3B). BP mainly included small molecule catalytic process, monocarboxylic acid catalytic process, and cellular lipid catalytic process, while CC comprised mitochondrial matrix, microbody, and peroxisomes. MF consisted of oxygen-induced activity, acting on the CH–CH group of donors, secondary active monocarboxylate transmembrane transporter activity, and monocarboxylate: medium symporter activity. In KEGG enrichment analysis (Fig. 3C), the PPAR signaling pathway (hsa03320), beta-Alanine metabolism (hsa00410), and Glycine, serine, and threonine metabolism (hsa00260) were found to play significant roles in ALF patients. We displayed the top 20 results in DO enrichment analysis using bubble plots. The target genes were primarily associated with fatty liver disease (DOID:9452), cholestasis (DOID:13580), and familial hyperlipidemia (DOID:1168) (Fig. 3D). Furthermore, we utilized STRING to explore the PPI network of the 191 ALF genes, and imported the data into Cytoscape to establish the PPI network (Fig. 3E, F), visually representing the relationships among these genes. The top 10 targets, including PCK2, CAT, GCDH, and GOT2, were screened according to the degree.

### 3.4 Identification of potential biomarkers of ALF based on machine learning algorithms

**Fig. 4:**
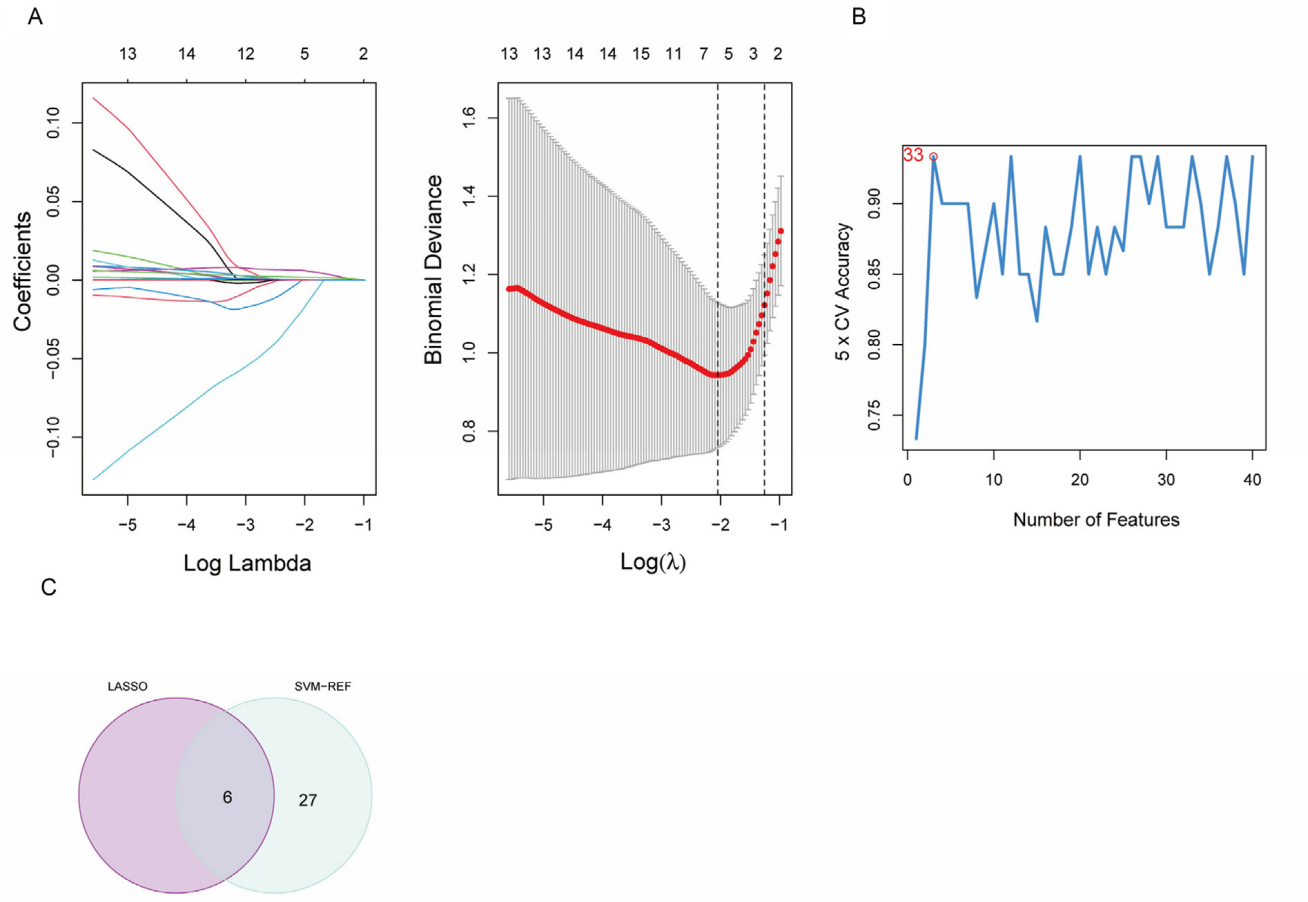
Selecting candidate ALF-specific genes by integrating LASSO and SVM algorithms. (A) In the LASSO model (λ) select the optimal variable of ALF (left) and the variable coefficient spectrum of specific genes in the ALF sample (right). (B) SVM-REF method for screening characteristic genes. (C) Venn diagram displaying candidate ALF feature genes by integrating the results of the two algorithms.

Using machine learning algorithms, the LASSO model successfully screened six genes (Fig. 4A) and the SVM model analyzed 33 genes (Fig. 4B). Using Venn diagrams (Fig. 4C), we identified six feature genes, *HORMAD2*, *WNT10A*, *ATP6V1E2*, *CMBL*, *ARRDC4*, and *LPIN2*, that were common to both screening methods. The *p*-value calculated from the hypergeometric distribution was 2.51 × 10^−12^.

### 3.5 Expression levels and diagnostic significance of potential biomarkers

**Fig. 5:**
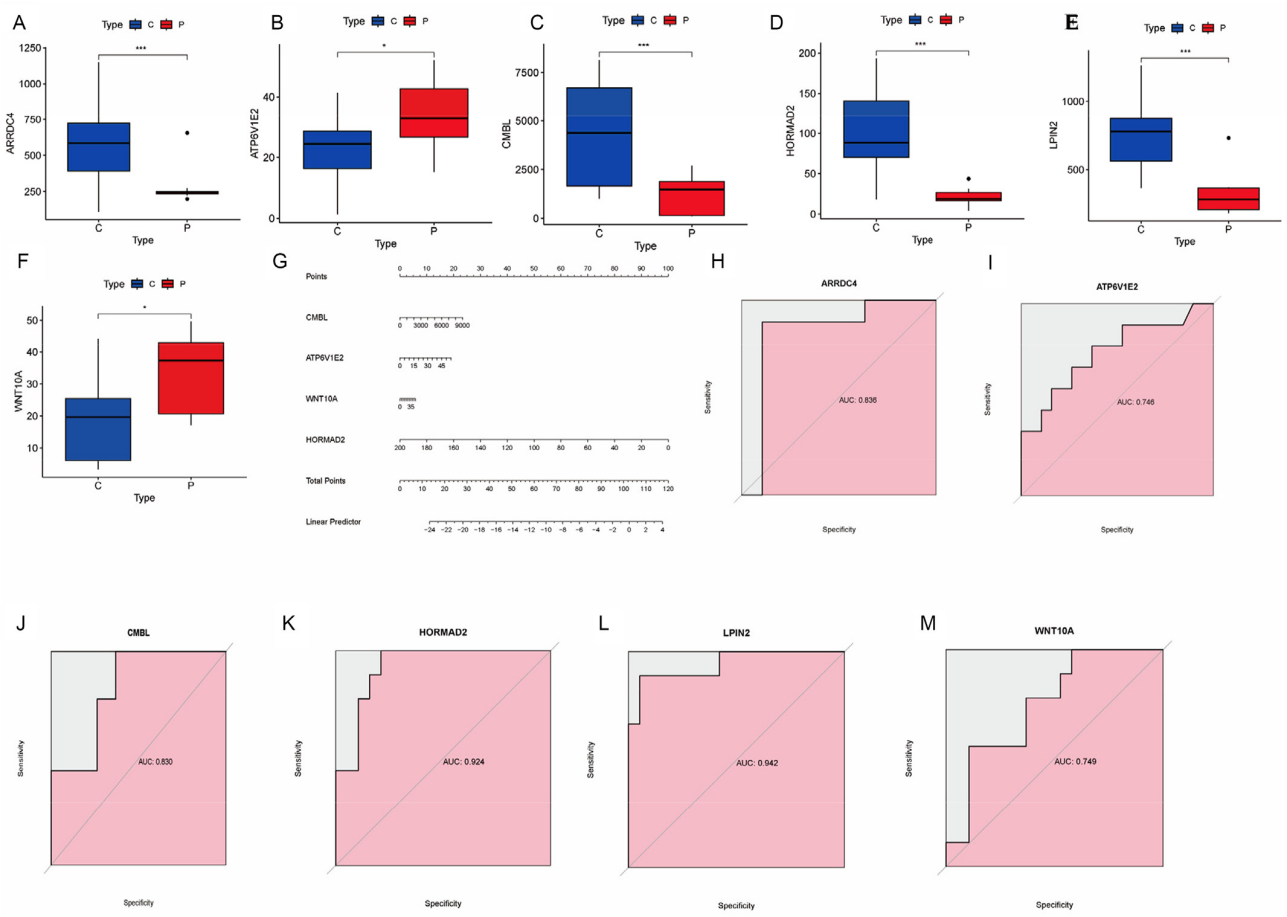
(A–F) Box plots of differential expression of characteristic genes in ALF and control groups. (G) ALF diagnostic column chart based on six genes. (H–M) ROC analysis of key genes in GSE14668. ∗*p* < 0.05; ∗∗*p* < 0.01; ∗∗∗*p* < 0.005.

The DEG box plot for GSE14668 revealed that *HORMAD2*, *CMBL*, *ARRDC4*, and *LPIN2* were downregulated in ALF patients compared with the control group, while *ATP6V1E2* and *WNT10A* were upregulated (Fig. 5A–F). By combining these six characteristic genes, a diagnostic column chart (Fig. 5G) was created, assigning each risk factor score. The total score derived from the sum of these indicators predicted the probability of ALF occurrence in each patient. ROC analysis showed AUC values for *HORMAD2*, *WNT10A*, *ATP6V1E2*, *CMBL*, *ARRDC4*, and *LPIN2* of 0.924, 0.749, 0.746, 0.830, 0.836, and 0.942, respectively (Fig. 5H–M), indicating their significant diagnostic values and suggesting that they may be promising biomarkers for ALF.

### 3.6 Further analysis of potential biomarkers

**Fig. 6:**
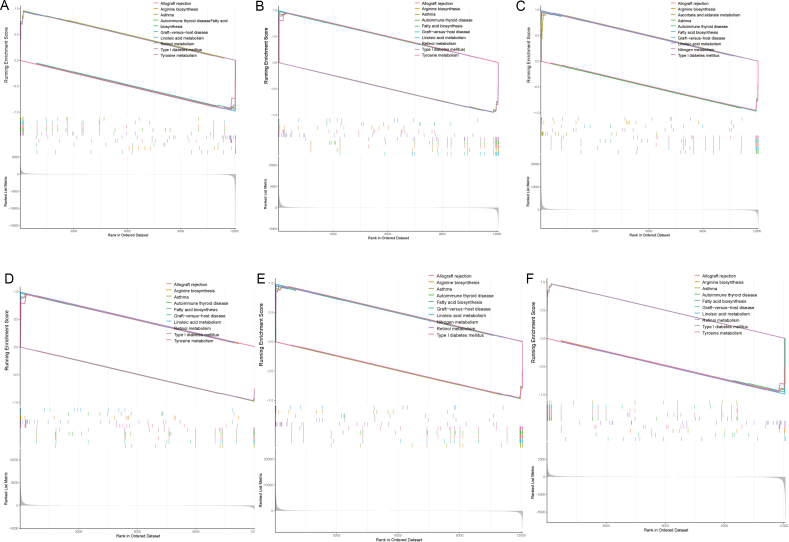
(A–F): Single gene GSEA of *HORMAD2*, *WNT10A*, *ATP6V1E2*, *CMBL*, *ARRDC4*, and *LPIN2*, showing the top 10 enriched pathways. (A) *HORMAD2*; (B) *CMBL*; (C) *ARRDC4*; (D) *LPIN2*; (E) *ATP6V1E2*; (F) *WNT10A*.

**Fig. 7:**
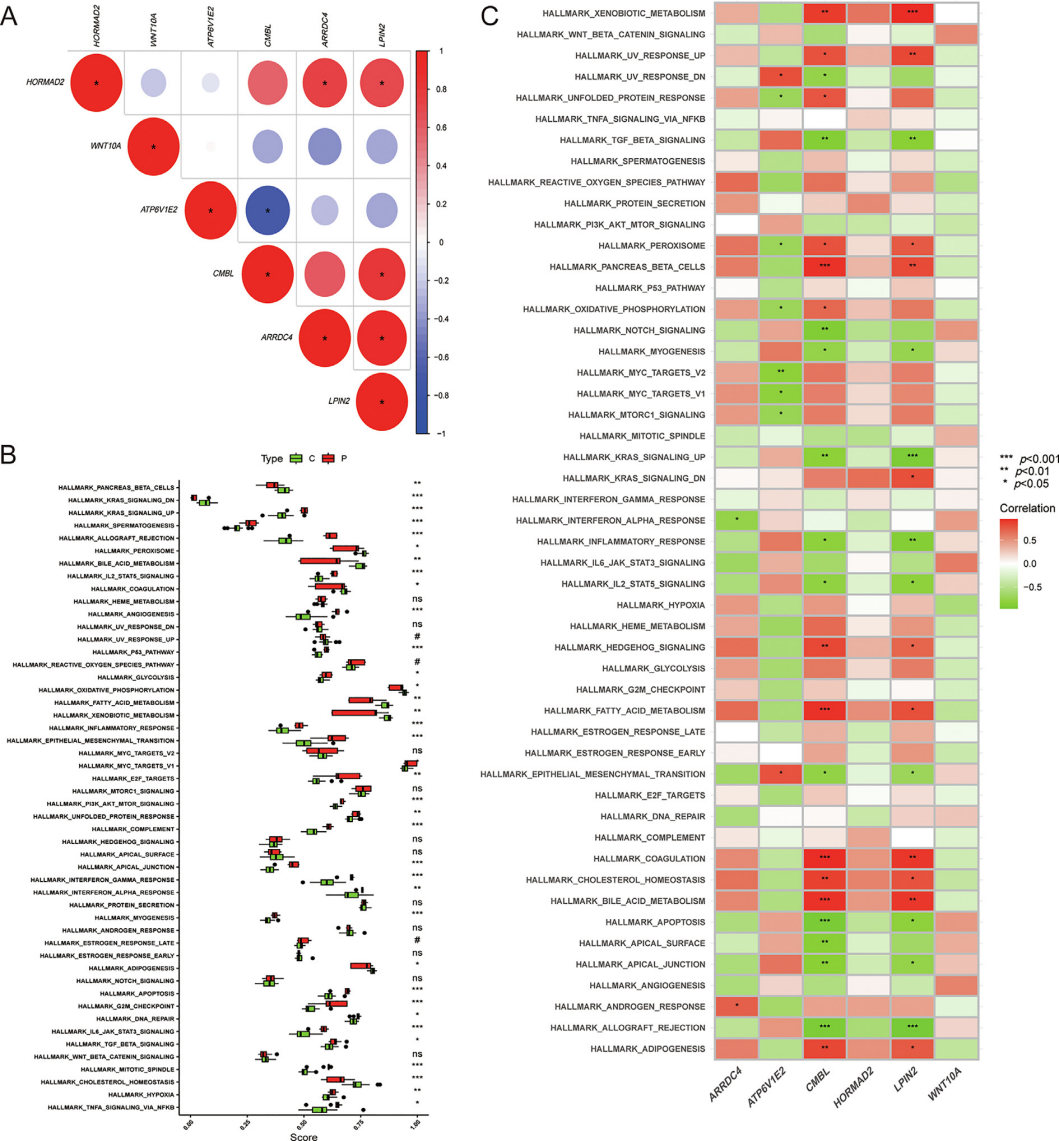
(A) Correlation between *HORMAD2*, *WNT10A*, *ATP6V1E2*, *CMBL*, *ARRDC4*, and *LPIN2*. (B, C) Functional analysis of ssGSEA marker gene set. ∗*p* < 0.05; ∗∗*p* < 0.01; ∗∗∗*p* < 0.005.

We determined the functions of potential biomarkers using GSEA. Single gene GSEA analysis revealed that *HORMAD2*, *WNT10A*, *ATP6V1E2*, *CMBL*, *ARRDC4*, and *LPIN2* were primarily involved in Allograft rejection, Arginine biosynthesis, Asthma, and Fatty acid biosynthesis (Fig. 6A–F). We then examined the correlations among *HORMAD2*, *WNT10A*, *ATP6V1E2*, *CMBL*, *ARRDC4*, and *LPIN2* (Fig. 7A). Notably, *CMBL*, *LPIN2*, and *ARRDC4* displayed negative correlations with *WNT10A* and *ATP6V1E2*, while *CMBL*, *HORMAD2*, and *ARRDC4* exhibited high coefficients with *LPIN2*. Furthermore, we conducted ssGSEA to analyze the associations between the six potential biomarkers and 50 marker gene sets, followed by a single gene correlation test. Most marker gene sets exhibited significant differences between ALF patients and normal controls (Fig. 7B). Additionally, both *CMBL* and *LPIN2* demonstrated strong correlations with HALLMARK_BILE_ACID_METABOLISM, HALLMARK_COAGULATION, HALLMARK_PANCREAS_BETA_CELLS, and HALLMARK_XENOBIOTIC_METABOLISM (*p* < 0.01) (Fig. 7C).

### 3.7 Distribution of immune cells

**Fig. 8:**
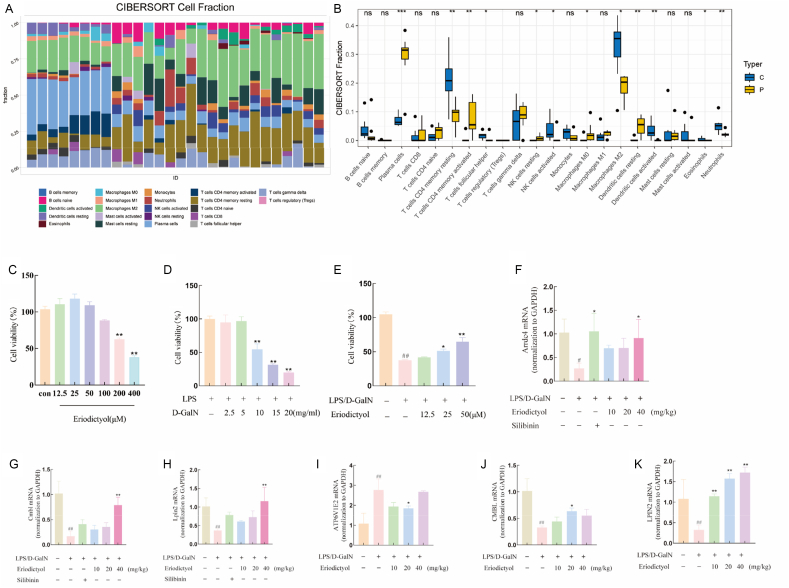
(A) Distribution map of immune cell proportions. (B) Differential distributions of immune cells between ALF and control samples. (C) Effects of different concentrations of eriodictyol on proliferation of HepG2 cells. (D) Effects of different concentrations of D-GalN and LPS on HepG2 cell proliferation. (E) Effects of combined therapy with different concentrations of eriodictyol and D-GalN (10 mg/mL) - LPS (1 μg/mL) on viability of HepG2 cells. (F–H) *In vivo* validation; relative expression levels of key gene mRNAs in liver tissues of mice. (I–K) *In vitro* validation; relative expression levels of key gene mRNAs in HepG2 cells. ∗, #*p* < 0.05; ∗∗, ##*p* < 0.01; ∗∗∗, ###*p* < 0.005.

We analyzed the immune microenvironment for the dataset using the CIBERSORT algorithm and generated distribution maps of 22 immune cell proportions for both ALF and control samples (Fig. 8A). Comparing the immune cell distributions between the ALF and control samples (Fig. 8B) revealed significantly higher infiltration of plasma cells, CD4 memory activated T cells, resting NK cells, M0 macrophages, and resting dendritic cells in the ALF group compared with the normal samples. In contrast, the infiltration of CD4 memory resting T cells, follicular helper T cells, activated NK cells, M2 macrophages, activated dendritic cells, eosinophils, and neutrophils were significantly lower in the ALF samples than in the normal samples. These differences in immune cell proportions between the two groups may be associated with the development of ALF.

### 3.8 Protective effect of eriodictyol on LPS/GalN-induced HepG2 cell damage

**Fig. 9:**
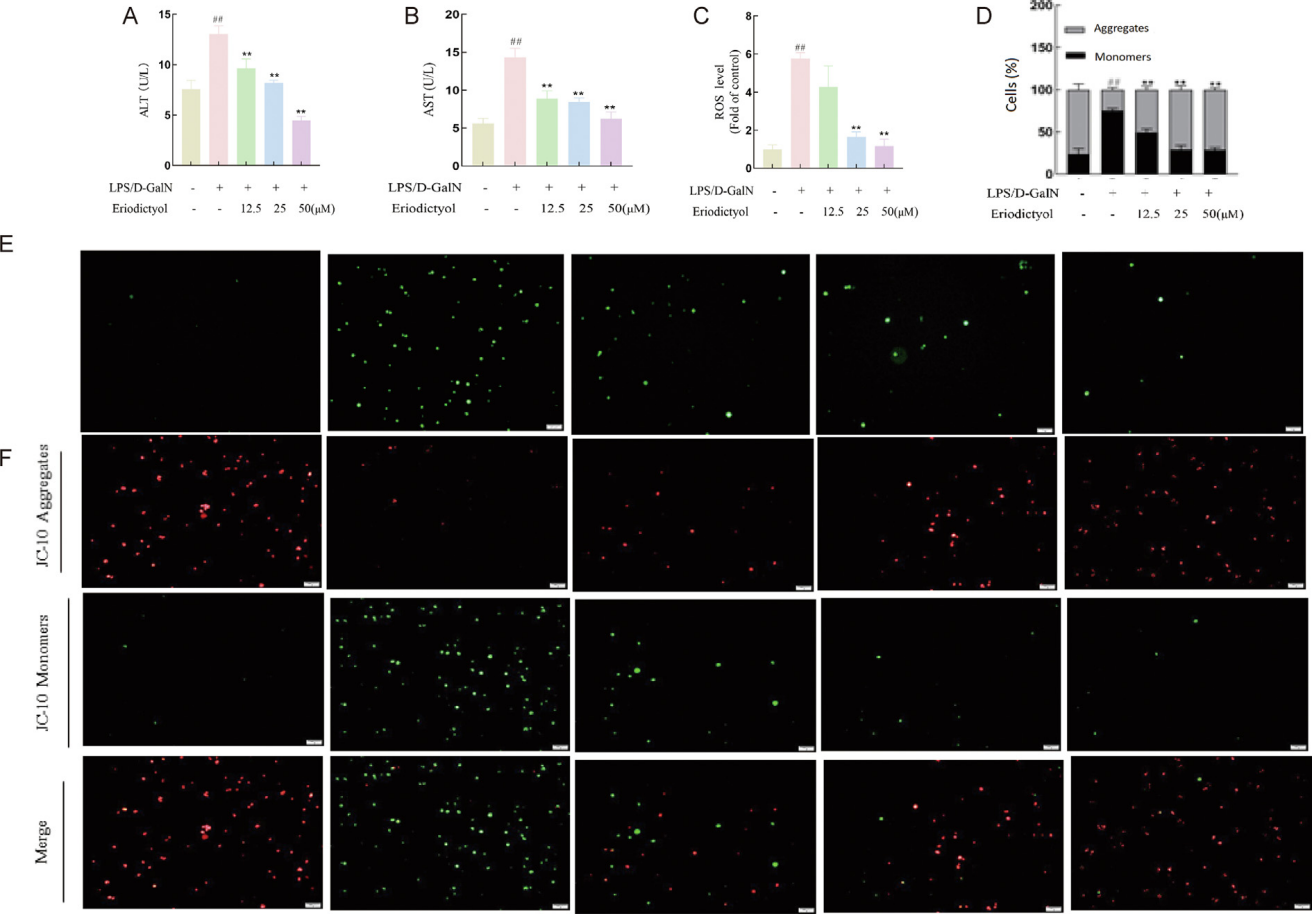
Protective effect of eriodictyol on LPS/GalN-induced damage in HepG2 cells. HepG2 cell culture supernatant was examined for levels of (A) ALT, (B) AST, and (C) ROS. (D) JC-10 red/green ratio was determined. (E) HepG2 cell ROS levels were observed under a fluorescence microscope. (F) JC-10 staining of HepG2 cells was observed under a fluorescence microscope (× 100). ∗*p* < 0.05; ∗∗, ##*p* < 0.01; ∗∗∗*p* < 0.005.

We assessed the extent of liver cell damage by measuring the ALT and AST activities in the cell supernatant. LPS/D-GalN triggered leakage of ALT and AST in HepG2 cells (*p* < 0.01) (Fig. 9A, B), while pretreatment with varying concentrations of eriodictyol significantly mitigated the release of ALT and AST induced by LPS/D-GalN (*p* < 0.01), in a dose-dependent manner. We also measured ROS levels to determine if eriodictyol could mitigate the oxidative stress induced by LPS/D-GalN in HepG2 cells. Compared with the control group, HepG2 cells treated with LPS/D-GalN exhibited a significant increase in ROS levels (*p* < 0.01), while pretreatment with eriodictyol significantly attenuated the intracellular ROS levels induced by LPS/D-GalN (*p* < 0.01) (Fig. 9C, E). The MMP serves as an indicator of both mitochondrial damage and early cellular apoptosis. We therefore investigated alterations in MMP in HepG2 cells subjected to oxidative damage induced by LPS/D-GalN, following pretreatment with varying concentrations of eriodictyol. Green fluorescence of JC-10 monomers was significantly increased in the model group, compared with the control group, accompanied by a decrease in red fluorescence of JC-10 polymers, indicating a reduction in MMP (Fig. 9D, F). Eriodictyol pretreatment dose-dependently inhibited the decrease in MMP induced by LPS/D-GalN, thus highlighting its protective effect.

### 3.9 *In vivo* and *in vitro* validation of potential biomarkers

Figure 8F–H presents the results of the *in vivo* validation experiments. *Arrdc4*, *Cmbl*, and *Lpin2* mRNA expression levels were significantly downregulated in liver tissue of ALF mice, compared with the control group (*p* < 0.05). Eriodictyol treatment reversed these effects to varying degrees, with significant upregulation (*p* < 0.05) in the high-dose eriodictyol group compared with the model group. We also conducted *in vitro* experiments on HepG2 cells treated with different concentrations of eriodictyol and D-GalN/LPS for 24 h. Eriodictyol 100–400 μmol/L inhibited HepG2 cell proliferation, with concentrations ≥200 μmol/L significantly suppressing HepG2 cell activity (Fig. 8C). The calculated IC50 of eriodictyol was 160.2 μmol/L. We investigated the inhibitory effect of D-GalN combined with LPS on the proliferation of HepG2 cells within the concentration range of 0–20 mg/mL, and subsequently selected subcellular toxicity concentrations of 10 mg/mL D-GalN combined with LPS, along with eriodictyol (12.5, 25, and 50 μmol/L) for further investigation. The activity of HepG2 cells was significantly decreased in the LPS/D-GalN-treated group compared with the control group (*p* < 0.01). Conversely, cell activity increased to varying degrees in the eriodictyol-treated groups, with 50 μM/L eriodictyol resulting in a significant increase (*p* < 0.01). Furthermore, qRT-PCR demonstrated a significant upregulation in *ATP6V1E2* mRNA expression levels (*p* < 0.01) in the LPS/D-GalN group compared with the blank group, while eriodictyol treatment led to varying degrees of downregulation, with 20 μM/L eriodictyol resulting in a significant decrease (*p* < 0.05) compared with the model group. Similarly, mRNA expression levels of *LPIN2* and *CMBL* were significantly downregulated in the LPS/D-GalN group compared with the blank group (*p* < 0.01), while eriodictyol dose-dependently reversed this effect, leading to upregulation of the relative mRNA expression levels of *LPIN2* and *CMBL* compared with the model group (Fig. 8F–H). These *in vivo* and *in* vitro results were consistent with the expression data observed in GSE14668.

## 4 Discussion

ALF, characterized by rapid hepatocyte death, can be triggered by various factors, including systemic inflammatory response syndrome [16,17]. Despite the significant impact of ALF however, its pathogenesis remains poorly understood, thus impeding the development of effective treatment approaches. Liver transplantation is currently the only viable option for end-stage liver failure and ALF [18]. This emphasizes the urgent requirement for a comprehensive understanding of the pathogenesis of ALF and the development of targeted therapies to enhance treatment outcomes for affected patients.

In this study, we used LASSO and SVM algorithms, both of which excel in handling high-dimensional data and feature selection. LASSO is a regularization technique that effectively performs variable selection, reduces model complexity, and enhances prediction accuracy. By imposing an L1 penalty, it encourages certain coefficients to become zero, thus achieving feature selection, which is particularly important when dealing with datasets with a large number of features [19]. On the other hand, SVM is a powerful tool for classification and regression, especially suited for high-dimensional data. SVM separates different classes of data points by finding the optimal hyperplane, exhibiting good generalization capability. By combining the strengths of LASSO and SVM, we can construct more accurate and robust predictive models, particularly for biomedical research [20]. Numerous studies have employed the combination of LASSO and SVM for gene selection and cancer classification, demonstrating their potential in bioinformatics [21]. Moreover, LASSO can be integrated with other machine learning algorithms to enhance model performance and interpretability [22]. This flexibility allows for the effective identification of the most predictive features when handling complex datasets, thus providing support for subsequent analysis and decision-making [23].

We initially obtained 352 DEGs by analyzing the gene expression profiling data for ALF patients and controls, to help identify potential diagnostic/prognostic biomarkers or therapeutic targets for ALF from highly correlated gene modules. Using the WGCNA data mining method, we further identified six key modules significantly associated with ALF development from the GSE14668 dataset. Notably, the turquoise module showed the strongest correlation with clinical ALF, and was thus the primary focus of subsequent analyses. The intersection of ALF genes and DEGs within the turquoise module yielded 191 differentially expressed ALF genes, which were subjected to enrichment analysis. Machine learning techniques were employed to handle the vast data and complex data relationships, and improve the identification of variables related to clinical outcomes, with enhanced predictive ability and robustness to data noise [24,25]. Machine learning algorithms are increasingly employed to create decision models for disease diagnosis and treatment [26]. In this study, we used the machine learning methods LASSO and SVM to screen six key genes (*HORMAD2*, *WNT10A*, *ATP6V1E2*, *CMBL*, *ARRDC4*, and *LPIN2*) associated with ALF. ROC analysis identified these six key genes as having high diagnostic accuracy for ALF. Additional analysis showed that expression levels of *LPIN2*, *CMBL*, *ARRDC4*, and *HORMAD2* were downregulated in ALF samples compared with normal samples, while expression levels of *ATP6V1E2* and *WNT10A* were upregulated in ALF samples. LPIN2, belonging to the LIPIN family, is an intracellular cytoplasmic protein exhibiting phosphatidic acid phosphate hydrolase activity, that plays a vital role in lipid metabolism. It also exerts regulatory effects on lipid intermediates in cell signal pathways and possesses transcriptional co-regulation capabilities [27,28]. LPIN2 has demonstrated involvement in various cellular processes, such as autophagy and inflammation, and it serves as a regulatory factor for gene expression [29,30]. Notably, Lpin2 has been shown to enhance insulin sensitivity by mitigating inflammatory responses [31]. The *CMBL* gene encodes the carboxymethylene butenol alkanolase homologue, belonging to the dienolactone hydrolase family as a cysteine hydrolase. A proteomics study by Yang et al. showed btoad expression of CMBL in various tissues, particularly in the liver and intestines [32]. ARRDC4, as an α-arrestin-like protein family member, possesses an inhibitory protein-like N domain, an inhibitory protein-like C domain, and a highly conserved polyproline motif at the C-terminus tail. Notably, ARRDC4 is recognized for its involvement in glucose metabolism, where it inhibits glucose uptake via its arrestin-like domain [33]. The functions of these key genes are consistent with the current findings.

Eriodictyol, a natural flavonoid from the flavanone subclass, is abundant in various medicinal plants, citrus fruits, and vegetables [34], and holds great promise for improving health. Numerous studies have demonstrated the beneficial therapeutic effects of eriodictyol on various liver conditions. Notably, Kwon et al. found that dietary eriodictyol alleviated obesity, fatty liver, insulin resistance, and inflammation in mice with diet-induced obesity [35]. Furthermore, Xie et al. demonstrated that eriodictyol had the potential to counter arsenic-induced toxicity, possibly by activating the Nrf2/HO-1 signaling pathway [14]. Eriodictyol also alleviated liver toxicity induced by acetaminophen and mitigated diet-induced liver steatosis in mice [15]. The similarity between LPS/D-GalN-induced liver injury and hepatitis B-induced liver injury means that the D-GalN-induced liver injury model is superior to the conventional model for examining liver injury pathogenesis [36]. In this study, we therefore utilized the LPS/D-GalN-induced ALF animal model to investigate the mechanisms of ALF treatment and potential therapeutic drugs. Eriodictyol was selected as the preventive measure in this model and ALT and AST were used as markers of hepatocellular damage [37]. Eriodictyol notably mitigated cell damage by significantly reducing the release of ALT and AST in HepG2 cells subjected to LPS/D-GalN treatment in this experiment. Intracellular ROS content serves as an indicator of oxidative stress levels [38]. We therefore further assessed the protective impact of eriodictyol on oxidative damage in HepG2 cells by analyzing ROS levels in cells damaged by LPS/D-GalN. The results demonstrated that eriodictyol significantly reduced the increased ROS levels induced by LPS/D-GalN. Mitochondrial damage can lead to oxidative stress in liver cells, which is closely associated with ROS generation. Additionally, the MMP reflects the extent of mitochondrial impairment and serves as a crucial early indicator of cellular apoptosis [39,40]. This study revealed that pretreatment with eriodictyol augmented the MMP in HepG2 cells induced by LPS/GalN, indicating its effectiveness in ameliorating mitochondrial dysfunction and inhibiting apoptosis, and further substantiating the robust antioxidant activity of eriodictyol. In summary, eriodictyol mitigated oxidative damage induced by LPS/D-GalN in HepG2 cells, thereby safeguarding liver cell integrity. qRT-PCR analysis revealed that eriodictyol upregulated the relative mRNA expression levels of *LPIN2*, *CMBL*, and *ARRDC4* in liver tissue, showing a protective effect against LPS/D-GalN-induced ALF. Eriodictyol also upregulated mRNA expression levels of *CMBL* and *LPIN2* at the cellular level, while downregulating mRNA expression levels of *ATP6V1E2*, thereby protecting against LPS/D-GalN-induced damage in HepG2 cells. These findings indicate the potential effectiveness of eriodictyol in ALF treatment. However, further research is warranted to explore its therapeutic effects more comprehensively. We also conducted CIBERSORT analysis to investigate immune cell function in ALF and revealed significant reductions in neutrophil and M2 macrophage infiltration in ALF patients compared with the control group, with no significant difference in the proportion of monocytes between the two groups.

There were also significant increases in the infiltration of plasma cells, CD4 memory activated T cells, and resting dendritic cells, consistent with previous findings [41,42], possibly linked to the onset and exacerbation of ALF. There is a growing consensus suggesting a close association between the imbalance of T cell subsets and the occurrence and progression of ALF [43]. Previous studies revealed that activated CD4 T cells, such as Th17 cells and Treg cells, played an important role in producing proinflammatory cytokines, leading to the escalation of systemic inflammatory responses. Consequently, excessive systemic inflammation contributes to the further advancement of ALF [44]. Moreover, activated monocytes can differentiate into macrophages, releasing chemokines, pro-inflammatory cytokines, and ROS, which in turn amplify pro-inflammatory signals and facilitate the accumulation of other inflammatory cells in the liver. These processes expedite the development of the systemic inflammatory response [45]. Yuan et al. demonstrated a close association between plasma cells and CD8 T cells with the infiltration of activated CD4 memory T cells [46].

This study had some limitations. We only explored DEGs using one ALF dataset, and the small sample size was a constraint in this study. Further large-scale validation is therefore warranted to confirm the findings.

## 5 Conclusions

In summary, this comprehensive study integrated bioinformatics analysis, machine learning techniques, and WGCNA to analyze a GEO ALF dataset. *HORMAD2*, *WNT10A*, *ATP6V1E2*, *CMBL*, *ARRDC4*, and *LPIN2* were identified as potential diagnostic biomarkers for ALF. Furthermore, cell experiments confirmed the protective efficacy of eriodictyol against LPS/D-GalN-induced ALF, and key gene expression alterations were validated by qRT-PCR. Additionally, our investigation delved into the intricate relationship between ALF and the immune system. These discoveries offer valuable insights, paving the way for further in-depth research to enhance the prognosis of patients with ALF.

## Funding

The authors did not receive support from any organization for the submitted work.

## CRediT authorship contribution statement

**Xinyan Wu:** Writing – original draft, Methodology, Investigation. **Xiaomei Zheng:** Writing – original draft, Software, Conceptualization. **Gang Ye:** Writing – review & editing, Validation, Resources.

## Acknowledgments

We thank Xiaoling Gong of Sichuan University for polishing this article.

## Declaration of competing interest

The authors state that the research was conducted without any commercial or financial relationships that could be perceived as potential conflicts of interest.

## Data availability statement

GSE14668 was downloaded from the Gene Expression Omnibus (GEO) (https://www.ncbi.nlm.nih.gov/geo/query/acc.cgi?acc=GSE14668).

## Ethics statement

This study was reviewed and approved by the Institutional Animal Care and Use Committee of Sichuan Agricultural University (NO. 20220710).

## Informed consent

Not applicable.
