# Supplementary material for: WGCNA combined with machine learning to explore potential biomarkers and treatment strategies for acute liver failure, with experimental validation
Source: ILIVER. 2024 Nov 13;3(4):100133. doi: 10.1016/j.iliver.2024.100133 (PMC12212670; doi:10.1016/j.iliver.2024.100133)
Supplement: Multimedia component 1 [file mmc1.docx]

## 1 Supplementary Method

## 1.1 Data acquisition

We conducted a targeted search of the GEO database (https://www.ncbi.nlm.nih.gov/geo/, accessed on March 2, 2024) with the keywords “acute liver failure” and “Homo sapiens”. Consequently, the dataset GSE14668, along with microarray matrix files, was downloaded. To ensure consistency, we employed the “sva” R package to normalize the expression matrix.

## 1.2 DEG analysis

R software (version 4.1.1) was utilized for data analysis. The gene expression profiling probe names were converted to gene names using the platform annotation file. DEG analysis was then conducted using the “limma” R package, with a significance threshold set at *p* < 0.05 and log fold-change >1. Visualization of the DEGs was carried out using heat maps and volcano maps.

## 1.3 Gene set enrichment analysis (GSEA)

We used GSEA to examine the disparity in biological function between liver failure and normal samples, and the biological function of risk signals associated with liver failure. This method determines the enrichment of a specific gene set in two distinct biological samples. Significance was assessed based on nominal *p* value <5% and false positive rate <25%.

## 1.4 WGCNA

We used the “WGCNA” R package to construct a co-expression network of all genes in ALF and normal samples, to identify a gene cluster highly associated with ALF. We then utilized the intersection of relevant genes from WGCNA and DEGs to determine the genes significantly linked to ALF. This process was accomplished using the Draw Venn Diagram online platform (http://bioinformatics.psb.ugent.be/webtools/Venn/, accessed on March 2, 2024).

## 1.5 Gene Ontology (GO), Kyoto Encyclopedia of Genes and Genomes (KEGG), and disease ontology (DO) analysis

We imported the common targets, specifically focusing on “*Homo sapiens”* and performed DO, GO enrichment analysis, and KEGG pathway analysis, using a significance threshold of *p*<0.05. These analyses aimed to explore genes significantly associated with ALF. The top 10 results from the GO, KEGG, and DO analyses were sorted and visually presented as bubble charts. The online tool used for these analyses was accessible at http://www.bioinformatics.com.cn/, accessed on March 3, 2024.

## 1.6 Protein-protein interaction (PPI) network construction

The common targets were submitted to the STRING database (https://string-db.org/, accessed on March 3, 2024) for further analysis. To ensure specificity, we conFig.d the species setting as *Homo sapiens*, and a confidence score >0.4 was chosen to construct an online PPI network. The resulting network was downloaded and saved in tsv file format for subsequent visualization. We imported this file into Cytoscape (https://cytoscape.org/, accessed on March 3, 2024) to generate a final network diagram. We then used the network analyzer tool (http://apps.cytoscape.org/networkanalyzer, accessed on March 3, 2024) to evaluate the degree distribution, clustering coefficient, and edge centrality, to gain insights into the potential interactions between significant genes associated with ALF at the protein level.

## 1.7 Screening core ALF genes based on machine learning

Biomarker screening was carried out using LASSO regression and SVM machine learning. LASSO regression, performed using the “glmnet” software package in R 4.1.1, facilitated variable screening while establishing a generalized model to identify significant predictive factors. SVM, as a linear classifier, employed the maximum interval principle to train samples through iterative processes, ultimately selecting the necessary features. The use of machine learning algorithms facilitated the screening of disease feature genes, and the subsequent analyses focused on the intersection of the two results to identify ALF feature genes.

## 1.8 Diagnosis and evaluation of core genes

The expression levels of the identified hub genes were compared between the ALF and control groups using the R packages “limma” and “ggpubr” The findings were represented visually using box plots. A ROC curve was generated and the area under the curve (AUC) with a 95% confidence interval was calculated. An AUC value approaching 1 signified a higher accuracy in model training.

## 1.9 Single gene GSEA and single sample GSEA (ssGSEA)

We evaluated the functional pathways associated with core genes using GSEA. The “limma” R package was utilized for functional enrichment pathway analysis of the marker gene set, to establish the link between core genes and the GSEA database marker gene set. We initially examined the functional differences between the ALF and normal groups, followed by further analysis to explore the discrepancies between core genes and the differentially enriched functions.

## 1.10 Analysis of correlation and expression differences of core genes

The correlation between core genes and the gene expression difference between ALF samples and normal samples were analyzed using the “limma” and “ggpubr” packages.
